# Structural Validity of an ICF-Based Measure of Activity and Participation for Children in Taiwan’s Disability Eligibility Determination System

**DOI:** 10.3390/ijerph17176134

**Published:** 2020-08-24

**Authors:** Ai-Wen Hwang, Chia-Feng Yen, Hua-Fang Liao, Wen-Chou Chi, Tsan-Hon Liou, Ben-Sheng Chang, Ting-Fang Wu, Lin-Ju Kang, Shu-Jen Lu, Rune J. Simeonsson, Tze-Hsuan Wang, Gary Bedell

**Affiliations:** 1 Graduate Institute of Early Intervention, College of Medicine, Chang Gung University, Taoyuan 33302, Taiwan; d93428001@ntu.edu.tw (A.-W.H.); lydiakang1003@gmail.com (L.-J.K.); 2 Department of Physical Medicine and Rehabilitation, Chang Gung Memorial Hospital, Linkou 33305, Taiwan; 3 Department of Public Health, Tzu Chi University, Hualien 97004, Taiwan; 4 School and Graduate Institute of Physical Therapy, National Taiwan University, Taipei 10055, Taiwan; 5 Department of Occupational Therapy, College of Medicine, Chung Shan Medical University, Taichung 40201, Taiwan; y6312002@gmail.com; 6 Department of Physical Medicine and Rehabilitation, Shuang Ho Hospital, Taipei Medical University, Taipei 11031, Taiwan; peter_liou@shh.org.tw; 7 Graduate Institute of Injury Prevention and Control, Taipei Medical University, Taipei 11031, Taiwan; 8 Department of Psychology, Soochow University, Taipei 11102, Taiwan; ben306@scu.edu.tw; 9 Graduate Institute of Rehabilitation Counseling, National Taiwan Normal University, Taipei 10610, Taiwan; tfwu@ntnu.edu.tw; 10 College of Medicine, School of Occupational Therapy, National Taiwan University, Taipei 10055, Taiwan; sjl470924@yahoo.com.tw; 11 School Psychology and Early Childhood Education, University of North Carolina at Chapel Hill, Chapel Hill, NC 27599-3500, USA; rjsimeon@email.unc.edu; 12 Graduate Institute of Physical Education, National Taiwan Sport University, Taoyuan 33301, Taiwan; wanda@thrct.org; 13 Department of Occupational Therapy, Tufts University, Medford, MA 02155-5539, USA; gary.bedell@tufts.edu

**Keywords:** participation, disability, measurement, children, adolescence

## Abstract

To assess activity and participation for children in Taiwan’s Disability Eligibility Determination System (DEDS), we developed a questionnaire, the Functioning Disability Evaluation Scale (FUNDES-Child), based on the Child and Adolescent Scale of Participation (CASP). The study follows a methodology research design to investigate the construct validity of the frequency and independence dimensions of FUNDES-Child 7.0. Two samples were randomly stratified from the databank of 13,835 children and youth with disabilities aged 6.0–17.9 years to examine structural validity by exploratory factor analysis (EFA, *n* = 4111, mean age of 11.3 ± 3.5) and confirmatory factor analysis (CFA, *n* = 4823, mean age of 11.4 ± 3.5)). EFA indicated a 4-factor structure for the frequency dimension (51.3% variance explained) and a 2-factor structure for the independence dimension (53.6% variance explained). The CFA indicated that the second-order factor structures of both dimensions were more parsimonious with adequate fit indices (Goodness fit Index, GFI; Normed Fit Index, NFI; Comparative Fit Index, CFI; and Tucker-Lewis Index, TLI ≥ 0.95, Root Mean Square Error of Approximation, RMSEA < 0.06). Results provide evidence that the participation part of FUNDES-Child 7.0 has acceptable structural validity for use in Taiwan’s DEDS. Utility of FUNDES-Child 7.0 in rehabilitation, welfare, and educational services needs further study.

## 1. Introduction

According to Taiwan’s People with Disabilities Rights Protection Act (promulgated in 2007), the local government in Taiwan was charged with developing a system to identify and classify disability and determine eligibility for provision of welfare services based on the framework of the International Classification of Functioning, Disability, and Health (ICF) and its child and youth version (ICF-CY) [1,2]. After five years of preparation, including the development and psychometric examination of measures for the eligibility determination and training of testers, the ICF-based Disability Eligibility Determination System (DEDS) was launched nationwide in July 2012. The disability identification is issued based on the results of the ICF-based disability evaluation by a medical team from authorized hospitals and needs assessment from the local social welfare department. The content of the disability evaluation includes physical examinations and assessments related to body function and structure (b/s) codes as well as activity and participation (A & P) components of the ICF and the ICF-CY. To assess the status of activity and participation in the ICF-based DEDS, Taiwan’s ICF taskforce group started to develop the Functioning Disability Evaluation Scale (FUNDES) from 2007 until it was formally implemented in July 2012 [3,4,5,6]. There is an adult version (FUNDES-Adult) and a child version (FUNDES-Child). To develop a reliable A & P measures in a timely manner, the FUNDES-Adult was developed and modified from the 36-item version of the WHO Disability Assessment Schedule 2.0 (WHODAS 2.0) [7] and the FUNDES-Child from the Child and Family Follow-up Survey (CFFS) [8,9,10].

The Functioning Scale of the Disability Evaluation System-Child version (FUNDES-Child) has been developed since 2007 as the tool for assessment of functioning (body function, activity and participation) and environmental factors in the DEDS for children aged 6–18 years. Details of the development and initial validation of FUNDES-Child have been described elsewhere [4,11,12,13]. In brief, the FUNDES-Child is an adapted version of the Child and Family Follow-up Survey (CFFS) [10] translated into traditional Chinese and back translated into English with approval and collaboration from the original author (Dr. Gary Bedell). The FUNDES-Child utilizes a proxy format in which parents or caregivers answer questions about their child’s activities in the previous 6 months. In keeping with the format used in the FUNDES-Adult version interview [3,5,6], flash cards with scoring options were used to assist parents in answering questions.

The participation part of FUNDES-Child has two dimensions, “independence” and “frequency”. “Independence” or “capability” describes the children’s ability to participate in age-expected life situations or to execute age-expected tasks or activities in daily settings as rated. “Frequency” refers to how often children engage in specific tasks or activities. Both dimensions are rated by children’s caregivers, who are familiar with the children as compared to same-age peers [14]. The items and response scale of the independence dimension of the participation section of FUNDES-Child was translated and modified from the Child and Adolescent Scale of Participation (CASP) [8,15], which was one part of the Child and Family Follow-up Survey (CFFS). The frequency dimension was designed by the Taiwan ICF team and added to each item of the participation section of FUNDES-Child [3,13] because the original CASP seemed to focus on the concept of ability (independence or capability of FUNDES-Child) and execute activities. The factor structure has been investigated for the independence dimension, but has not been examined for the frequency dimension [8,11]. The development of the FUNDES adult and child versions were completed in 2012, the formal implementation year of the current DEDS. Therefore, we used the data of the FUNDES-Child 7th version (FUNDES-Child 7.0) of the Taiwan Databank of People with Disabilities, which begins in 2013 and is relatively stable and mature, compared to the previous version, to examine the factor structures of both the independence and frequency dimensions in this study. Understanding the internal factor structure helps to manage datasets with large numbers of observed variables that are thought to reflect a smaller number of underlying/latent variables.

Psychometric properties have been examined in previous FUNDES-Child versions [12,13,16,17,18]. Internal consistency of the independence and frequency dimensions were adequate to excellent for the total score of the participation section of FUNDES-Child (Cronbach’s alpha = 0.81–0.96) [13]. Test-retest and inter-rater reliabilities were also found to be adequate to excellent (ICC = 0.85–0.99) [11,13]. Independence and frequency scores based on parent/caregiver reports about the child’s school participation were not significantly different from scores based on teacher reports [13]. Known-groups validity evidence was found for the participation independence dimension of FUNDES-Child based on significantly different scores between children with less severe and more severe intellectual disabilities [12]. In addition, prior confirmatory factor analysis yielded a two-factor structure contributing to 64.1% of explained variance for the independence dimension based on a sample of about 200 children with disabilities (ages 6–18) in Taiwan. The two factors were named as the “daily living” and “social/leisure/communication” [11].

Due to the need for cultural adaptation and the practical demands of nationwide application, revisions to FUNDES-Child have been ongoing and are based on feedback from field testers and experts as well as results from data analyses conducted nearly every year. Several versions of the FUNDES have been developed. The cultural adaptations included that the 19th item is about “using transportation to get around in the community (e.g., to and from school, work, social or leisure activities) (driving vehicle or using public transportation), we used bicycle to replace vehicle due to the act regulation for an age limit to drive a vehicle is different from that of other countries) and the item 20th is about “work activities and responsibilities (e.g., completion of work tasks, punctuality, attendance and getting along with supervisors and co-workers). We treat the objects in vocational schools who were working because of the internship and teaching cooperation classes as students in the Taiwan education system. The other reason is about the format of FUNDES-Child, which should be consistent with FUNDES-Adult [3,5]. The construct validity of the two-factor structure of the independence dimension of the participation part of FUNDES-Child has been examined and supported by exploratory factor analysis. The structure factor of the frequency dimension has not yet been examined. The construct validity of a measure, especially the structural validity, is important [19]. The purpose of this study was to examine the structural validity of participation part (both the independence and frequency dimensions) of FUNDES-Child. All processes of FUNDES-Child development are listed in Table 1.

## 2. Materials and Methods

### 2.1. Design

This cross-sectional study was part of a larger national survey conducted in Taiwan by the Taiwan ICF team [6]. The present study was approved by the Research Ethics Committee of the Hualien Tzu Chi Hospital, Buddhist Tzu Chi Medical Foundation (IRB104-04-A;IRB107-46-B) and Joint Institutional Review Board Taipei Medical University (TMU- Joint Institutional Review Board), Taiwan. The deidentified data were retrieved from the Taiwan Databank of People with Disabilities, which included 14,835 children and youth (aged 6.0–17.9 years) who received the DEDS assessment in 201 authorized hospitals from November 2013 to January 2015 [3,12,16,17,20]. All children and youth were assigned a diagnosis with specific codes of the International Classification of Disease, 9th Revision, Clinical Modification (ICD-9-CM) (http://www.cdc.gov/nchs/icd/icd9cm.htm) to be eligible for the DEDS.

### 2.2. Participants

Deidentification of information of the seventh version of the FUNDES in the Taiwan Databank of People with Disabilities was used in this study [20]. The FUNDES 7th version was used to collect information related to “activity and participation” of people with disabilities from July 2013 to January 2015 when the current ICF-based disability evaluation system was launched. There is one item pertaining to work in FUNDES-Child participation part. Therefore, if the student was not working, this item would be not applicable, and parents/caregivers would only complete 19 of the 20 items in this section. Given that the majority of individuals in the databank were not working, this study only examined 19 items. Therefore, individuals who were employed were excluded from the total sample (*n* = 14,835), resulting in data on 13,835 children and youth aged 6.0–17.9 years left for factor analyses.

The 13,835 children and youth from the larger sample were then randomly allocated into 3 smaller samples of roughly the same size: sample 1 (*n* = 4111), sample 2 (*n* = 4824), and sample 3 (*n* = 4900). Sample 1 was used for exploratory factor analyses (EFA), and sample 2 and 3 were to be used for the confirmatory factor analyses (CFA) and potential modifications needed for model fit. Because the CFA results of sample 2 showed good model fit, it was unnecessary to conduct CFA in sample 3.

### 2.3. Procedures

Individuals with information in the Taiwan Databank of People with Disabilities were evaluated via face-to-face interview by physicians and a qualified physical therapist, occupational therapist, social worker, clinical psychologist, or nurse practitioner in the authorized hospitals. The databank included a record of demographic characteristics (including personal factors), assessments of the individual’s body function and body structures, activity and participation functioning, and some environmental conditions.

The severity level of a person’s impairment was determined in the medical examination stage of the DEDS. Relevant ICF body function/structure categories for specific diagnoses were coded by physicians trained in using a 0- to 4-point qualifier scale (no problem = 0, mild = 1, moderate = 2, severe = 3, and profound = 4). A final summative severity level was determined based on decision rules for combining levels of severity among the individual body function/structure codes [21]. There were 8 types of impairment in the DEDS based on the 8 Body Functions Chapters of the ICF.

### 2.4. Measures

FUNDES-Child has 79 items, including 4 parts: physical and emotional health (5 items), participation (40 items), the child and adolescent factors inventory (15 items), and the child and adolescent scale of environment (19 items). The participation part was the focus of this study, which includes 20 items in each of the following dimensions: independence and frequency [4,16]. The participation part measures children’s extent of participation frequency and independence in home, school, and community life situations and related activities in the previous 6 months compared to same-age peers. The 20 items are divided into 4 domains: home participation, school participation, community participation, and home and community living activities. Domain 1, home participation, has 6 items (item 1–6) to assess participation in the home setting and includes social, play, or leisure activities; chores; self-care activities; communication; and moving around at home. Domain 2, neighborhood and community participation, has 4 items (item 7–10) to assess activities including social, play, or leisure activities; structured events; moving around; and communicating with others in community; Domain 3, school participation, has 5 items (item 11–15) to assess activities including educational (academic) activities; social, play, or leisure activities; moving around; and using educational material in schools. Domain 4, home and community living activities (HCLA), has 5 items (item 16–20) to assess activities including household tasks, shopping and managing money, managing schedule, using transportation to get around, and work activities and responsibility in home and in the transition to community [15].

The items are rated on a four-point scale for two dimensions, independence (independent (0), supervision or mild assistance (1), moderate assistance (2), and full assistance (3)) and frequency (age-expected frequency (0), somewhat less frequent than expected for age (1), much less frequent than expected for age (2), and did not participate (3)) [4]. A “not applicable” response is allowed when the proxy perceives that the child’s peers in the community would not be expected to participate on specific items. All items were rated under the assumption that children used assistive devices as usual. For example, an item would be rated as 0 (independent) if the child could participate in an activity with his/her existing devices and without others’ assistance. Higher scores indicate greater restriction in participation, reflecting more difficulty, less independence, and lower frequency of participation. The differences between two dimensions of the “activities and participation” components, frequency and independence of FUNDES-Child, could be used to understand the possible impacts of environmental factors [14]. The purpose of this study was, thus, to examine the factor structures of the independence and frequency dimensions of FUNDES-Child 7.0.

### 2.5. Data Reduction and Statistical Method

About 30% of the data from all 13,835 children were randomly selected as sample 1 (*n* = 4111) for EFA. About a half of the remaining dataset was selected as sample 2 (*n* = 4824) for CFA. Statistical analyses and EFA were performed using SPSS 20.0 (IBM SPSS Statistics, Chicago, IL, USA, 2016). Since most observed item distributions violated normality assumptions and were inter-correlated, we used the iterative principal axis factoring followed by oblique promax rotation [22]. Factorability of items was examined by the Bartlett test (α was set at 0.05) and the Kaiser–Meyer–Olkin (KMO) measure of sampling adequacy. A value of KMO greater than 0.6 is tolerable for EFA [23]. The number of factors was decided by multiple methods including eigenvalues > 1 and scree tests. Factor loadings ≥ 0.3 were considered salient loadings [22]. The extracted latent factors were then named based on conceptual interpretation of the items. For each factor, the average score, standard deviation, range, and internal consistency were reported. The internal consistency was assessed by Cronbach’s alpha coefficient. Values between 0.70 and 0.95 are considered adequate [24].

Based on the results of EFA, the first-order model solutions of the frequency and independence dimensions were examined by maximum-likelihood CFA using sample 2 with AMOS 20.0 (IBM, Inc., Armonk, NY, USA, 2012). Considering the presence of multivariate non-normal data, the Bollen–Stine bootstrap methods were applied [25]. The second-order models for both dimensions were also examined by CFA. The second-order model could be a more parsimonious model with all first-order latent factors loaded onto one second-order factor. To assess model fit, fit indices with their cutoff criteria (goodness-of-fit index (GFI) ≥ 0.95, normed fit index (NFI) ≥ 0.95, comparative fit index (CFI) ≥ 0.95, Tucker–Lewis index (TLI) ≥ 0.95, root mean square error of approximation (RMSEA) < 0.06) were used [24,26].

The Chi-square difference test was used for comparing first-order and second-order models. The target coefficient (T), which is the ratio of the Chi-square of the first-order model to the Chi-square of the higher-order (more restrictive) model, was used to evaluate whether the first- or second-order model is preferable for the data [27]. A value of T close to 1 suggests that a second-order model is preferable.

## 3. Results

### 3.1. Characteristics of the Samples

Demographic data and health characteristics of the sample are presented in Table 1. Among the 13,835 children, 66% were male and had a mean age of 11.4 (SD = 3.5) years. The majority of children (91%) were classified as having a single type of impairment, with Chapter 1 (mental functions/structures of the nervous system) being the most common type (88%), followed by Chapter 7 (5%, neuromusculoskeletal and movement-related functions/structures) of the ICF coding system. Children with multiple disabilities comprised less than 9% of the sample. The severity level of disability of 60% of the sample was classified as mild and with children able to live independently in their communities. Similar characteristics were found among the EFA and CFA subsamples and the larger sample (Table 2).

### 3.2. Exploratory Factor Analyses and Internal Consistency

For the 19 items of the frequency dimension, the KMO measure of sampling adequacy was 0.912, and the Bartlett test of sphericity was statistically significant (*p* < 0.001). Thus, the data were suitable for EFA. The initial EFA extracted five factors with eigenvalues above 1.0 and explained 54.9% of the variance. However, the eigenvalue of the fifth factor was 1.01, with only two items (items 1 and 6) loading on it. Therefore, the 4-factor model was retained with 51.3% variance explained (Table 3). The factors were named as “daily living participation frequency” (4 items), “mobility participation frequency” (5 items), “learning participation frequency” (6 items), and “community participation frequency” (4 items). The factors correlation matrix in Table 2 showed moderate correlations across all factors (r = 0.45–0.59).

For the 19 items of the independence dimension, the KMO measure was 0.951 and the Bartlett test was significant (*p* < 0.001). The initial EFA extracted three factors (eigenvalues = 9.71, 1.37, and 1.01) and explained 57.3% of the variance. However, the Scree plot indicated a two factor solution. Therefore, the 2-factor model was retained with 53.6% variance explained (Table 4). The factors were named as ‘daily living independence’ (10 items) and ‘social participation independence (9 items). The correlation coefficient between the 2 factors was 0.75.

The summary scores of the four frequency factors and two independence factors of the FUNDES-Child participation part were the sum of actual ratings for items within a given factor divided by the maximum total ratings for items within that factor, and converted to a 0–100 scale. As an example, a summary score for the 4-item factor of daily living participation frequency might be ratings of 3_,_ 3, 2, 1 = 9 divided by four maximum ratings of 3 = 12 for a score of 9/12 converted to 75 on a 0–100 scale. The range of all factor summary scores was all from 0 to 100. The higher score means higher restriction and lower frequencies. The average summary scores and standard deviations of the four frequency factors were 50.2 ± 24.0 (daily living participation frequency), 29.2 ± 21.7 (mobility participation frequency), 37.4 ± 22.1 (learning participation frequency), 56.6 ± 25.3 (community participation frequency), with Cronbach’s α of 0.776, 0.774, 0.835, and 0.833, respectively. For the two independence factors, the mean scores were 36.3 ± 22.3 (daily living independence) and 40.6 ± 24.6 (social participation independence), with Cronbach’s α of 0.909 and 0.924, respectively.

### 3.3. Confirmatory Factor Analyses

**Figure 1:**
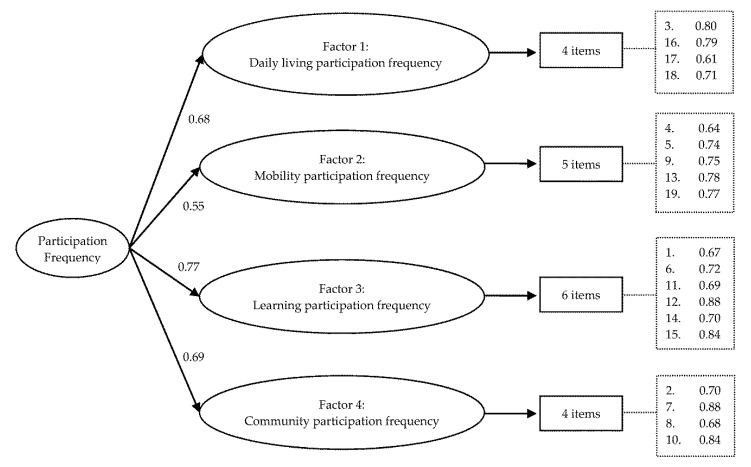
Second-order confirmatory factor analysis model of the frequency dimension of the participation part of the Functioning Disability Evaluation Scale-Child version (*n* = 4823).

As the EFA suggested a four-factor solution for the frequency dimension, a CFA using sample 2 was conducted with a first-order 4-factor model allowing the four latent factors to correlate freely (model F-1). All standardized factor loadings were greater than 0.51, and the fit indices indicated a good fit (Table 4). The second CFA using a more parsimonious second-order 4-factor model (model F-2) was performed. All fit indices showed a good fit. Comparing the first-order model with the second-order model using the likelihood ratio test resulted in a non-significant Chi-square test (χ2(9) = 8.4, *p* = 0.49). In addition, the target coefficient (T) of 0.95 supported the second-order construct. Therefore, the more parsimonious second-order 4-factor model was preferred (Figure 1). All 19 items have factor loadings greater than 0.61 on their corresponding factors, supporting the construct validity of the frequency dimension (Figure 1).

**Figure 2:**
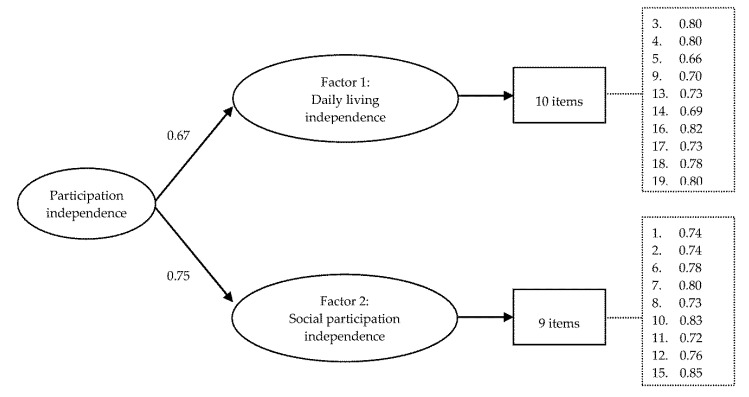
Second-order confirmatory factor analysis model of the independence dimension of the participation part of the Functioning Disability Evaluation Scale-Child version (*n* = 4823).

For the independence dimension, a CFA using sample 2 with 2 factors emerging from the EFA was conducted. The two latent factors were allowed to correlate freely (model I-1). All standardized factor loadings were greater than 0.61, and the fit indices indicated a good fit (Table 5). For the second-order model (model I-2), all fit indices showed a good fit and the likelihood ratio test yielded a non-significant Chi-square test (χ2(1) = 0.4, *p* = 0.53). The target coefficient (T) of 0.998 also supported the second-order construct. As shown in Figure 2, all items have factor loadings greater than 0.66.

## 4. Discussion

Participation is one of the most significant outcomes of rehabilitation, social, and educational interventions [28]. Using a large nationwide Disability Eligibility Determination System (DEDS) sample, the results of this study provided evidence of construct (structural) validity and internal consistency of the participation part of FUNDES-Child for children and youth aged 6 to 18 years old. The results of this study confirmed that the 19 items of the independence dimension of the participation part of FUNDES-Child had distinct factor loadings on the two derived factors (daily living independence and social participation independence) with one higher-order construct of participation independence. These two derived factors are the same as those found in a previous Taiwan study based on a sample of 200 children with various disabilities ages 6–18 years [11]. A new finding from this study suggested a four-factor solution for the frequency dimension along with one higher-order construct of participation frequency. The items loading on each of the four factors reflected the following domains: daily living participation frequency, mobility participation frequency, learning participation frequency, and community participation frequency. The factor summary scores calculated on the basis of the factor structure are important for disability practices, research, and policies.

There were 6 items loaded in the learning participation frequency factor of FUNDES-Child. Four items are activities related to communication, social, leisure, and education in school settings, two items are activities related to communication, social, and leisure in home settings. It is possible that the factors that influence interaction frequency with other persons is similar for children with disabilities, regardless of whether they happen at home or at school. One of our previous studies demonstrated that Taiwan’s school and home settings provided relatively sufficient support for children with disabilities to participate [14].

There are some similarities in the findings on the independence dimension in this study and earlier research. The factor structure of the initial (English language) version of the CASP that was the forerunner of FUNDES-Child has been studied using different samples. As mentioned, the initial CASP has the same items and as is likely assessing components of the independence dimension of FUNDES-Child, has been explored by using EFA in one sample of 60 children with acquired brain injury (ABI) aged 3–27 years [9], in 313 children with varied diagnoses (56% children with ABI) aged 3–27 years [8], in 409 youth with varied diagnoses and aged 11–18 years [29], and in 926 children with traumatic brain injury and isolated arm injury aged 0–18 years [30]. The results of the US samples indicated a two-factor solution [9], three-factor solution [8,29] or four-factor solution [30]. Except for one subsample of Golos and Bedell’s study (2016), many items had cross loading on two or three factors in U.S. samples. Recently, a one-factor structure of the German version of the CASP was reported [31]. Therefore, the two-factor structure of the CASP in two Chinese samples as well as the independence dimension of FUNDES-Child in the study of Hwang et al. and this study are stable with more distinct factor loadings [11].

The possible reasons for somewhat different factor structures among the studies include differences in samples, influence of cultural and language differences, and testing procedures between the initial US CASP and Chinese FUNDES-Child. In addition, the more stable and distinctive factor structure found in two Chinese samples might be due to the standardized interview procedure used in collecting participation data. As mentioned before, the FUNDES-Child and FUNDES-Adult questionnaires were developed for the purposes of the ICF-based Disability Eligibility Determination System (DEDS) to respond to the regulation mandated by the People with Disabilities Rights Protection Act. To increase the utility of the questionnaires in the DEDS and fit Taiwan’s culture and to ensure confidence of applicants and the government officials, the FUNDES team gradually examined their reliability and validities during implementation, and modified the FUNDES-Child and FUNDES-Adult to have a similar format and fit Taiwan’s culture [3,5,6]. For example, flash cards with scoring options were used to assist parents in answering questions in FUNDES-Child.

The distinct factor loading on two derived factors of the independence dimension allows for the calculation of two factor summary scores: daily living independence score and social participation independence score. Most items of daily living independence domain come from the home setting and home and community living activities, and are related to mobility and daily care tasks, while items of social participation independence domain are from community and school setting, and are related to communication and social tasks. Similarly, the participation dimension yields four factor summary scores: daily living participation frequency score, mobility participation frequency score, learning participation frequency score, and community participation frequency score. The items of learning participation frequency and community participation frequency are the same as daily living independence. The items of daily living participation frequency and mobility participation frequency are almost the same as social participation independence except item 4 (home meals with family). For the frequency dimension, item 4 cross-loaded on Factor 2 (mobility participation frequency) and on Factor 1 (Daily living participation frequency), with factor loading of 0.447 and 0.370, respectively. As mentioned before, each frequency item was modified from the independence item of the CASP. Item 4 of the CASP is “self-care activities (e.g., eating, dressing, bathing, combing or brushing hair, using the toilet)”. Such self-care activities are basic needs for every child with disabilities and they are involved in those activities almost every day at home in Taiwan. However, for children with disabilities, parents tend to feed them before family meals. We thought that children’s experiences of attending family mealtime and interacting with family members are important home participation activities. Therefore, we designed the frequency dimension of item 4 as “how frequently the child has meals with family”. Thus, children with greater mobility restriction participation may participate in family meals less frequently.

The results of this study also provide internal consistency evidence related to the FUNDES-Child participation part. The strong internal consistency evidence found for the two subscales in the independence dimension is similar to what has been found in prior studies [8,11,29,30,32]. Overall, these results suggest that the items were inter-related and related to the factor and the whole scale. The other important benefits of our findings of the factor summary scores for disability practices, research, and policies are (1) to allow exploration of which aspects of the gaps of “independence” and “frequency” may be due to environmental factors, which can help us to address the environmental issues to affect child’s development; (2) our new factor summary scores in Factor 1 “daily living independence” and Factor 2 “social participation independence” can help policymakers to allocate social welfare resources. Family support and resources should be inducted more for subjects who get higher summary scores in daily living or to figure out the barriers of social participation in the subgroups with higher scores in the Social participation domain.

As mentioned before, in Taiwan’s DEDS system, the proxy report of FUNDES-Child has been used. However, the scores between the parent/caregiver report version and youth self-report version of the CASP or of FUNDES-Child are significantly different [8,13,29,33,34]. Children and parents do not only show differences in the perception of participation, they also identify different priorities for participation goals in the individualized educational plan [13,35]. Therefore, stakeholders need to consider their specific research or practice purposes in determining whether to use one or both versions.

Taiwan has used an ICF-based measure of activity and participation successfully in the DEDS. The first stage of needs assessment in Taiwan’s DEDS applies the scores for FUNDES to determine the social welfare supports related to mobility restriction, the necessity of accompaniment (that means there is one attendant that can accompany the person with a disability with free tickets to participate in social activities if the activities are paid), and RehabBus. The application of FUNDES by clinicians or social welfare service providers in order to understand what are the restrictions and to enhance the social participation for people with disabilities has also been proposed [4,5,13,34]. In Taiwan, the ICF framework has been applied in early intervention and special education services [34,35,36,37]. However, FUNDES-Child requires additional testing and validation. We hope the main theme of ICF—enhancing the full participation of people with disabilities in society—could be reached through the application of FUNDES-Child.

There are a few limitations of this study that must be noted. First, although the large sample size of this study provided sufficient statistical power, the majority of children in the sample had intellectual disabilities. Thus, further study would be needed for children who were less represented in the sample.

Second, this study did not use a theoretical construct of participation for children with disabilities. In ICF, the definition of participation is “involvement in a life situation” [1]. However, there is no clear international consensus on the participation construct in pediatric settings. Imms et al. (2017) have proposed a family of participation-related constructs (fPRC), indicating that participation has two essential components: attendance, defined as “being there” and measured as frequency of attending and/or the range or diversity of activities; and involvement, the experience of participation [38]. The two dimensions of the participation part of FUNDES-Child, independence and frequency, belong to different components of the fPRC. The frequency dimension belongs the attendance component and the independence dimension may be seen as belonging to performance competence component of the fPRC. Further studies are needed to examine the utility of the summary factor scores of these two dimensions in disability determination systems as well as rehabilitation and special education services.

## 5. Conclusions

The FUNDES-Child participation part was adapted to Taiwanese culture and includes an independence and frequency dimension across the home, preschool, school, and community settings for children and youth. The results from the exploratory and confirmatory factor analyses provide evidence for the structural validity of FUNDES-Child based on samples drawn from the larger Taiwan population of children ages 6.0–17.9 years with disabilities. The evidence was strong for the independence 2-factor solution with strong internal consistency of the two domains, showing promise for use as subscales, since the findings provide preliminary psychometric evidence to inform the application of FUNDES-Child to assess children’s “activity and participation” independence or capabilities and frequencies of attendance and, also, to inform how scores can be used for different purposes in Taiwan.

## Figures and Tables

**Table 1:** The process of the Functioning Scale of the Disability Evaluation System-Child version (FUNDES-Child) tools development.

FUNDES-Child Versions	The Date of Design Completion and Start Training Program	Subscales and Dimensions or Illustrates
Has been developing	2010–2011 Concept formation and design The tool was being developing	CASP has been bi-directionally translated. Four subscales: (1) home participation, (2) community participation, (3) school participation, and (4) home and community living activities. One independence (capability) dimension.
FUNDES-Child 5.0 *	2011/09	Four subscales: (1) home participation, (2) community participation, (3) school participation, and (4) home and community living activities. Two dimensions of participation: “independence” and “frequency”. The format of FUNDES-Child should be consistent with FUNDES-Adult. Add flash cards. To develop the scoring based on ICF.
FUNDES-Child 6.0	2012/07-12	Developing the manuals. The adult and children’s training programs and versions are combined in the same manual.
FUNDES-Child 7.0	2014/06-12-2017	To separate adult and children’s manual and add evidence of the reliability and validity of FUNDES 7.0 (adult and child versions) into the manual. Due to the need for cultural adaptation and the practical demands of nationwide application, revisions to FUNDES-Child have been ongoing based on feedback from field testers and experts as well as results after 2017.

* To match the naming of the FUNDES-Adult version.

**Table 2:** Characteristics of the study sample.

Characteristics			All Participants *n* (%)	Sample 1 for EFA ^2^	Sample 2 for CFA ^2^
			*n* = 13,835	*n* = 4111	*n* = 4823
Gender					
	Male		9101 (65.8)	2705 (65.8)	3184 (66.0)
	Female		4734 (34.2)	1406 (34.2)	1639 (34.0)
Age (years)					
	6–9		4733 (34.2)	1444 (35.1)	1644 (34.1)
	10–13		4636 (33.5)	1357 (33.0)	1604 (33.3)
	14–17		4466 (32.3)	1310 (31.9)	1575 (32.6)
	Mean age		11.4 ± 3.5	11.3 ± 3.5	11.4 ± 3.5
Number of impairments identified ^1^					
	Single type of disability		12,644 (91.4)	3751 (91.2)	4411 (91.4)
	Types of disability based on b/s Chapter				
		Chapter 1	11,083 (87.7)	3288 (87.7)	3848 (87.2)
		Chapter 2	438 (3.5)	136 (3.6)	169 (3.8)
		Chapter 3	107 (0.8)	32 (0.9)	36 (0.8)
		Chapter 4	210 (1.7)	64 (1.7)	70 (1.6)
		Chapter 5	14 (0.1)	7 (0.2)	3 (0.1)
		Chapter 6	30 (0.2)	11 (0.3)	12 (0.3)
		Chapter 7	584 (4.6)	161 (4.3)	206 (4.7)
		Chapter 8	13 (0.1)	3 (0.1)	7 (0.2)
	Others (chromosome or gene et al.)		165 (1.3)	49 (1.3)	60 (1.3)
	Two types of disabilities		1033 (7.5)	311 (7.6)	360 (7.5)
	More than three types of disabilities		158 (1.1)	49 (1.2)	52 (1.1)
Disability severity level					
	Mild		8241 (59.6)	2464 (59.9)	2889 (60.0)
	Moderate		3949 (28.5)	1171 (28.5)	1367 (28.3)
	Severe		1104 (8.0)	325 (7.9)	393 (8.1)
	Profound		541 (3.9)	153 (3.7)	174 (3.6)
Living situation					
	Independent living in community		8042 (58.1)	2455 (59.8)	2743 (56.9)
	Assisted living in community		5616 (40.6)	1609 (39.1)	2016 (41.8)
	Not living in community		177 (1.3)	47 (1.1)	64 (1.3)

^1^ Type of disability was classified using the International Classification of Functioning, Disability, and Health (ICF) Body Functions and Structures (b/s) Chapter. Chapter 1: mental functions/structures of the nervous system; Chapter 2: sensory functions (b2)/the eye, ear, and related structures (s2); Chapter 3: voice and speech functions/structures; Chapter 4: functions/structures of the cardiovascular, hematological, immunological, and respiratory systems; Chapter 5: functions/structures of the digestive, metabolic, and endocrine systems; Chapter 6: genitourinary and reproductive functions/structures; Chapter 7: neuromusculoskeletal and movement-related functions/structures; Chapter 8: functions/structures of the skin and related structures. Each child might have more than one type of disability. ^2^ Abbreviations: EFA, exploratory factor analysis; CFA, confirmatory factor analysis.

**Table 3:** Factor loadings of the frequency dimension of FUNDES-Child by exploratory factor analysis (*n* = 4111).

Item No. and Name		Factor ^1,2^			
		1	2	3	4
3. Home: Family chores, responsibilities, and decisions		**0.821**	−0.077	0.012	0.047
16. HCLA: Household activities		**0.810**	−0.005	−0.070	0.061
18. HCLA: Managing daily schedule		**0.403**	0.322	0.105	−0.092
17. HCLA: Shopping and managing money		**0.356**	0.279	−0.071	0.127
13. School: Moving around		−0.180	**0.639**	0.278	0.022
9. Community: Moving around		−0.104	**0.617**	−0.207	0.462
5. Home: Moving around		0.030	**0.552**	0.138	−0.041
19. HCLA: Using transportation to get around		0.181	**0.480**	−0.063	0.025
4. Home: meals with family		0.370	**0.447**	0.116	−0.169
15. School: Communicating with other children and adults		−0.057	−0.018	**0.715**	0.201
11. School: Educational activities with classmates		−0.048	0.115	**0.701**	−0.095
12. School: Social, play, and recreational activities with classmates		−0.063	0.047	**0.633**	0.143
14. School: Using educational materials and equipment		−0.007	0.367	**0.479**	−0.136
6. Home: Communicating with other children and adults		0.176	−0.009	**0.470**	0.173
1. Home: Social, play, or leisure activities with family members		0.182	−0.039	**0.431**	0.136
7. Community: Social, play, or leisure activities with friends		−0.024	−0.028	0.028	**0.874**
10. Community: Communicating with other children and adults		0.013	0.063	0.096	**0.696**
8. Community: Structured events and activities		0.111	0.156	−0.069	**0.579**
2. Home: Social, play, or leisure activities with friends		0.064	−0.162	0.265	**0.533**
Variance explained	(total = 51.3%)	11.9%	12.8%	14.2%	12.4%
Factors inter-correlation coefficients	Factor 1		0.55	0.54	0.46
	Factor 2			0.59	0.45
	Factor 3				0.56

^1^ Salient factor loadings (>0.3) are shown in bold. ^2^ Factor 1 = daily living participation frequency; Factor 2 = mobility participation frequency; Factor 3 = learning participation frequency; Factor 4 = community participation frequency; FUNDES-Child = Functioning Disability Evaluation Scale-Child version; HCLA = home and community living activities.

**Table 4:** Factor loadings of the independence dimension of FUNDES-Child by exploratory factor analysis (*n* = 4111).

Item No. and Name		Factor ^1,2^	
		1	2
16. HCLA: Household activities		**0.821**	−0.027
4. Home: Self-care activities		**0.812**	−0.048
3. Home: Family chores, responsibilities, and decisions		**0.704**	0.074
5. Home: Moving around		**0.670**	−0.035
18. HCLA: Managing daily schedule		**0.647**	0.064
13. School: Moving around		**0.625**	0.105
19. HCLA: Using transportation to get around		**0.610**	0.018
17. HCLA: Shopping and managing money		**0.602**	0.118
9. Community: Moving around		**0.471**	0.243
14. School: Using educational materials and equipment		**0.425**	0.300
7. Community: Social, play, or leisure activities with friends		−0.125	**0.911**
10. Community: Communicating with other children and adults		−0.004	**0.821**
2. Home: Social, play, or leisure activities with friends		−0.078	**0.791**
15. School: Communicating with other children and adults		0.054	**0.759**
12. School: Social, play, and recreational activities with classmates		0.140	**0.648**
6. Home: Communicating with other children and adults		0.124	**0.648**
8. Community: Structured events and activities		0.154	**0.611**
11. School: Educational activities with classmates		0.186	**0.539**
1. Home: Social, play, or leisure activities with family members		0.230	**0.517**
Variance explained	(total = 53.6%)	26.6%	27.0%
Factors inter-correlation coefficients	Factor 1		0.75

^1^ The salient loadings were usually recognized when they are beyond 0.3, which are shown in bold. ^2^ Factor 1 = daily living independence; Factor 2 = social participation independence; FUNDES-Child = Functioning Disability Evaluation Scale-Child version; HCLA = home and community living activities.

**Table 5:** Model fit indices for the frequency and independence dimensions of FUNDES-Child ^1^ by confirmatory factor analyses (*n* = 4823).

CFA Model	BSχ^2^	df	GFI	NFI	CFI	TLI	RMSEA
Frequency dimension							
F-1. First-order 4-factor model	175.2	149	0.996	0.996	0.999	0.996	0.006
F-2. Second-order 4-factor model	183.6	158	0.996	0.996	0.999	0.997	0.006
Independence dimension							
I-1. First-order 2-factor model	190.6	151	0.997	0.997	0.999	0.994	0.007
I-2. Second-order 2-factor model	191.0	152	0.997	0.997	0.999	0.994	0.007

^1^ FUNDES-Child = Functioning Disability Evaluation Scale-Child version; BSχ2 = Bollen–Stine Chi-square; GFI = goodness-of-fit index; NFI = normalized fit index; CFI = comparative fit index; TLI = Tucker–Lewis index; RMSEA = root mean square error of approximation.

## Abbreviations

DEDS	Disability Eligibility Determination System
FUNDES	Functioning Disability Evaluation Scale
CASP	Child and Adolescent Scale of Participation
HCLA	home and community living activities
